# Olfactory enrichment using a maternal pheromone improved post-weaning pig performance and behavior

**DOI:** 10.3389/fvets.2022.965370

**Published:** 2022-11-08

**Authors:** Courtney Archer, Arlene Garcia, Madelyn Henderson, John J. McGlone

**Affiliations:** ^1^Laboratory of Animal Behavior, Physiology and Welfare, Texas Tech University, Animal and Food Sciences, Lubbock, TX, United States; ^2^Texas Tech School of Veterinary Medicine, Amarillo, TX, United States

**Keywords:** pig, pheromone, weaning, maternal, behavior

## Abstract

The post-weaning environment in commercial pig nursery buildings is typically devoid of both the sow and her semiochemicals. Among other factors, the loss of maternal odors may contribute to post-weaning stress. In this work, we report four studies (studies 1-4) using 192 weaned pigs to evaluate the effects of a maternal pheromone (MP) containing Myristic Acid and Skatole on weaned pig behavior and performance. Study 1 examined behavior among weaned pigs with a focus on lying, aggression and feeding behaviors. All studies used body weight gain and the percentage of pigs that lost weight the first 7 days after weaning as key response variables. The MP stimulated early feeding behavior and reduced aggressive behaviors among weaned pigs (*p* < *0.01*). In study one, pigs were over 4 kg heavier 16 weeks post-weaning when the MP was present for 48 h post-weaning compared to control pigs (*p* = *0.05*). The three other studies used a 7-days bioassay to assess the best dose and form of delivery of the MP among weaned pigs. The single measure that responded uniformly was the percentage of pigs that lost body weight in the first 7 days after weaning. Among control pigs in four separate studies, 25% of the weaned pigs in each study lost body weight by 7 days after weaning, while in 3 of the 4 bioassay studies, 0% of MP-exposed pigs lost weight after weaning (one study had 6% of pigs losing body weight with the MP). This MP effect is both highly statistically significant and, if found on commercial farms, would have a large positive economic benefit. Simultaneously, having fewer pigs losing weight and exhibiting less aggressive behavior will improve the health and welfare of weaned pigs. Providing biologically-relevant olfactory enrichment can improve pig health and welfare.

## Introduction

Commercial piglets are weaned at an earlier age than their natural setting in the wild. While feral pigs generally practice gradual weaning over a period of weeks (1), piglets in commercial swine units are abruptly weaned at 17 to 24 days. In the United States, older weaning ages are found among some niche-market products. Even certain countries with niche-markets value weaning at 28 or 35 days of age, but this is still earlier than when feral pigs wean their piglets. The weaning age selected by commercial pork producers is set by the economics of the breeding herd, not the biology of the pig. Sow milk production begins to decline after about 21 days of lactation which makes it a logical weaning age. Earlier weaning can improve the numbers of piglets produced per sow per year on farms. Weaning at an early age (before the natural weaning age) may cause production and behavioral problems while enhancing farm profitability (2).

Weaning is stressful to piglets due to the number of changes within their environment. The environment transitions from a familiarity of litter mates and maternal presence to strange odors and foreign piglets filling the post-weaning pen. The change from maternal milk to solid dry pellets can cause starvation, malnourishment, and reduced growth rate (1–3). The first week of weaning is the most influential, as the impact of weight gain/loss immediately post-weaning drives later average daily gain and the continued improvement or decline of the piglet (4). The absence of the maternal semiochemicals in the nursery pens leads to negative biological results among weaned piglets (5). Typically, weaned pigs average about the same body weight 7 days post-weaning as their initial weaning weight. This is because some pigs lose weight shortly after weaning (6) while some gain weight and therefore, on average, it requires 7 days for weaned pigs to regain the weight they lost the first few days after weaning.

During lactation, piglets nurse about 20 times a day, nearly hourly. After weaning, dry feed is presented to them usually in a continuous manner. The feed form is different (solid grain-soy rather than milk), and the behavior required to consume dry feed is different than suckling. In addition, the lack of maternal odors leads to piglet confusion and a delay in solid feed consumption.

Piglets learn the odor of its mother by 12 h of life (7). While an earlier putative maternal pheromone was marketed, it was not a commercial success. The synthetic maternal pheromone was not as good as biological fluids, so we sought to determine if a maternal-neonatal pheromone could be discovered. Many of the molecules found on the lactating sow's skin secretions (and feces) are also found in pregnant sows. If the molecules are present in adult pigs, it may be an odor signature, not a pheromone that changes piglet behavior and physiology. Our laboratory then discovered, when we fed sows the same diet during gestation and lactation in the same amounts, that 2 molecules (myristic acid and skatole) appear in higher concentrations during lactation, but not at other times in the sow's life cycle (5). In an initial report, we showed that applying this maternal-neonatal pheromone (MP) on and around the feeder, promoted about a 30% increase in feeding and reduction in aggressive behaviors (5). At that point, we had not refined the most effective dose or delivery methods.

A MP is an important semiochemical from a female directed toward her offspring. Some of the first research conducted with maternal pheromone was in rats in the 1980's (8, 9). The MP we investigated here was identified in 2020 as the semiochemical within the feces (and skin secretions) of sows that create behavioral and performance effect among weaned piglets (5) Synthetic maternal pheromone research has shown positive results in reducing aggression and skin lesions in weaned pigs (10). One recent study that added the MP to lactating sow enrichment tassels (strips of material for sows to play with peri-farrowing) showed that litters with MP near the time of farrowing decreased mortality in piglets (11).

The goals of these studies were to (1) confirm that this novel MP stimulated post-weaning performance, reduced aggression, and increased feeding, and (2) develop a simple bioassay to determine the extent to which the MP can stimulate weight gain and reduce fighting when the dose and delivery methods were improved. We report a simple bioassay to test concentrations and delivery methods of MP or similar technologies. We also report in a longer-term study that tracked pig performance into the finishing phase showed potential benefits of this new concept. Small improvements in post-weaning performance can result in larger weight gain later in life.

## Materials and methods

Four studies were conducted at the Texas Tech University (TTU) research farm to determine a method of application of the maternal pheromone to newly weaned piglets. One study kept pigs on study until finishing to examine the long-term effects of MP on weaned pigs. The maternal pheromone was applied to the treatment feeders in each study to identify its effects on growth, behavior, and performance.

### Animals, housing and statistics

All studies were approved by the TTU Animal Care and Use Committee prior to the start of the work (IACUC # 19104-12). Studies were conducted at the TTU swine unit which is an 80-sow farrow-to-finish operation near the TTU campus. The farm has two, 16-crate farrowing rooms and 2 nursery rooms. Each room has separate ventilation with 100% fresh air entering. Pigs were fed standard corn-soy-vitamin-mineral diets. All piglets had ad libitum access to feed *via* a five-hole feeder and a single nipple drinker per pen. Room temperatures were set at about 32 C at weaning and dropped about 2 C per week to accommodate the thermal needs of the piglets. Piglets were housed four per pen with wire or plastic flooring providing 3.2 m^2^ of total floor space. Piglets were derived from PIC Camborough sows and PIC Duroc boars.

Each study randomly distributed weaned piglets and blocked them by litter, sex, and weight to treatments. Piglets were weaned at 21 days of age with an average weight of about 5.5 kg (specific data reported for each study). During the studies, we had no pig deaths. The pigs were considered high health with no major infectious diseases.

The pen was the experimental unit for all studies, while also controlling for litter, sex and body weight. Each study was balanced in that there was an equal number of experimental units per treatment group. Each pen contained two castrated males and two females. Pig gender does not have a large effect on pig performance or behavior at this age.

A total of 192 weaned pigs were evaluated. Numbers of pigs and pens are given for each study below.

General Linear Models were used to analyze and summarize the parametric data. The first model evaluated each of the three treatments, compared with the control group. When we had a control and 2 forms or doses of MP, we also performed linear contrasts to compare control with the combined MP treatments. When more than two treatments were present, the predicted different test was used to separate means in each study. All statistics were performed by SAS software (SAS, Cary, NC) or Microsoft Excel.

An experiment-wise or a meta-analyses was used to assess multiple dose and form of delivery studies to see if an overall effect of MP treatment could be assessed relative to the control group. Also, these small range-finding studies are useful to determine an adequate sample size best to use for the bioassay. At this early stage in development of this technology, mean differences in the face of large variation are difficult to find significant differences where they may be present. Therefore, for studies 2 through 4, we looked only for mean differences, not statistically significant differences.

When all studies were combined, we performed a power test to estimate the sample size needed for a *P* < 0.05 mean effect with an alpha of 0.80 for a single study to be statistically significant. This information can establish both the sample size needed for future bioassays or longer-term studies.

### Studies

#### Study 1—Sponge vs. bead application

Rather than having people spray feeders with the MP as in our previous work, we sought ways to apply it to the feeder with minimal labor. To investigate this, we first examined two technologies: sponges and beads. Treatments included replicate pens containing either; a control (with nothing added), two sponges hung over the feeder onto which the MP was poured at times zero and 24 h post-weaning, or sodium alginate beads containing MP. We examined 4 replicate pens for each of three treatments (Control, Sponge-MP and Beads-MP).

**Figure 1:**
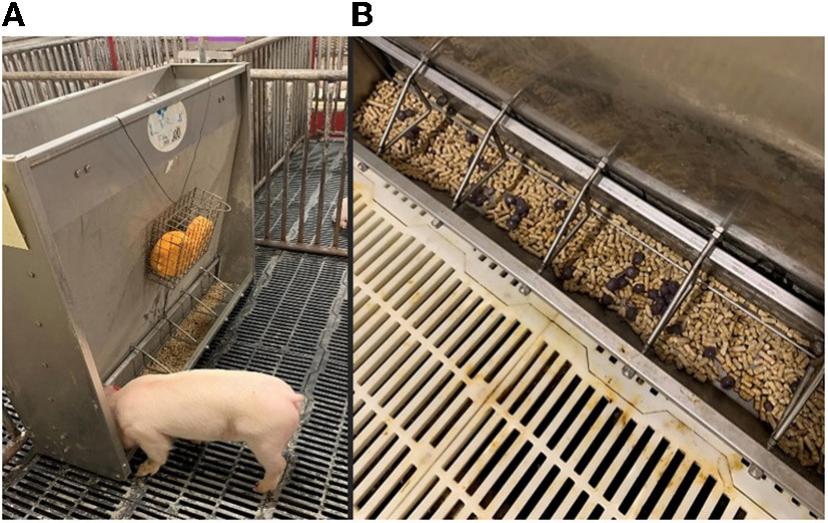
These photos show the nursery pens used in this study. 100 mL of MP was added to the sponges and hung over the feeder just out of the reach of the pigs [**(A)**, left photo]. 100 g of beads were added on top of the nursery feed [**(B)**, right photo].

Figure 1A shows the placement of the sponge treatment group. A metal cage was mounted to the feeder, roughly 5 cm above the feed trough. Each cage held two round sponges which released the odor of the maternal pheromone. The pheromone was applied at time 0 (the start of the study) and again after 24 h. Each sponge treatment pen received 100 mL of pheromone at each application period. Piglets were not able to touch the sponges. Piglets could not reach the MP in the sponges; thus, they were as close to the feed as possible without pig direct interaction.

The second treatment can be seen in Figure 1B. Beads were made of food grade sodium alginate (12) with infused pheromone in the beads. Beads were dyed with food coloring to easily assess the presence or absence of the beads in the feed troughs. Each pen received 100 mL of pheromone evenly distributed through 100 grams of beads. The amount of pheromone in each bead is unknown; the 100 mL (as was poured on the sponges) was encapsulated in the beads. Piglets had full access to chew, root, eat and interact with the pheromone-infused beads.

The maternal pheromone in Study 1 was diluted in 70% isopropyl and blue dye with over-the-counter food coloring. A stock solution of the MP with 10 μg concentration was diluted into 100 mL of 70% isopropyl to decrease the concentration to 1 ppm for the treatment groups. This dilution was poured directly on to the sponges or infused into the beads before applying to the feeders. A total of 1,600 mL of pheromone was used throughout Study 1.

Performance (weight in kg) recordings for this study were collected at time of weaning as well as 7 days, 4-, 8-, 12-, and 16-weeks post-weaning (even though piglets were exposed to the MP only the first 48 h after weaning).

Behaviors were recorded for the first 48 h of the study (2 days post-weaning) to identify the impact treatments had on aggression, feeding, and lying behaviors in weaned piglets relative to the control group. Behaviors are defined in Table 1. All video observers were validated prior to data collection. To be a validated observer, that person's data on at least 10 videos had to be a statistically identical mean as the standard/control observer. In addition, a regression analysis confirming similar data when the values are low or high had to produce an R-value of over 0.90 for the person to be considered validated. Behaviors were continuously recorded for individual pigs and pens using BORIS software. BORIS is an online free, open-sourced software system that allows coding for computer-based review of animal behaviors from recorded videos or live observations (13).

**Table 1:** Definitions of behaviors summarized by validated observers.

**Behavior**	**Definition**
Aggression	Two piglets or more, interacting nose to nose with aggressive inclinations. Aggression was not included for inter-pen aggressive behaviors.
Feeding	Piglet's head was fully submerged within feed trough. Shoulders forward on the piglet could not be seen due to placement inside the feeder.
Lying	All four ligaments were down, with elbows and knees touching the floor. Laying on sternum or side counted as lying behavior.

Statistical analyses are the same as described above. This study had three treatments; however orthogonal linear contrasts were used to compare control with the combined MP treatments. This study showed that the MP response was clear 1 week after weaning based on body weights, ADG and the percentage of pigs that lost body. Thus, the 7-days bioassay was developed and used in the remaining studies to refine the formulation. In this bioassay, key measures were ADG the first 7 days after weaning, and the percentage of weaned pigs that lost body weight the first 7 days after weaning.

#### Study 2—Myristic acid methyl ester vs. myristic acid

The methyl ester form of fatty acids is more volatile than fatty acids alone. In this case, myristic acid is less volatile than methyl ester myristic acid (14). The information acquired from Study 1 illustrated that applying the pheromone to a sponge or beads over the feeder caused increased growth rates. Therefore, study 2 delivered the same sponge application method as Study 1. A total of 48 piglets were blocked by litter, gender and weight and were randomly distributed into 6 pens assigned to control, Myristic Acid (standard formula), or MAME (Methyl Ester formula) treatments. Pheromone treatments were assessed by examining the first week's body weight gain and the percentage of pigs that lost weight in the 7 days after weaning. Control groups received no pheromone application. Body weights were collected at time of weaning and 7 days later. The percent of pigs losing weight was also summarized. The placement of the pens in the nursery allowed for space between treatment groups to minimally contaminate the control piglets with MP (and certainly if control pigs had any MP exposure, it was well–below an effective dose. Key measures in this bioassay were ADG the first 7 days post-weaning and the percentage of weaned pigs that lost body weight the first 7 days after weaning.

#### Study 3—Low dose vs. high dose

**Figure 2:**
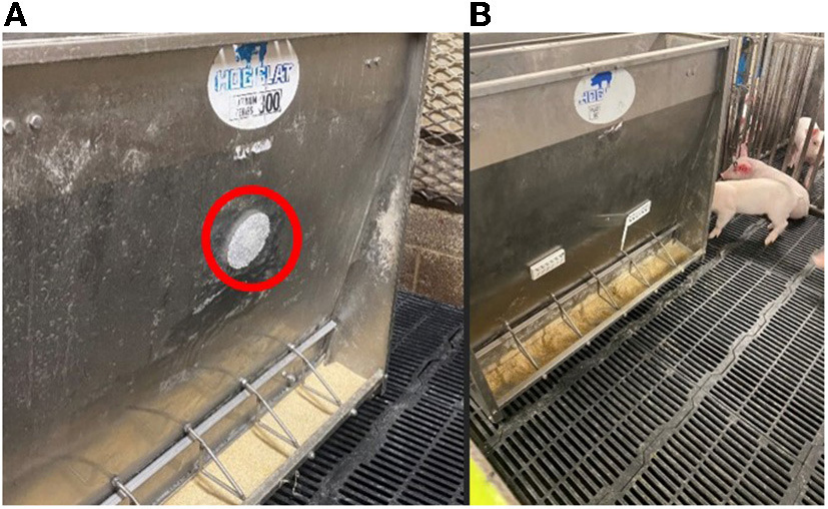
Photo of the “cookie” attached to the nursery feeder just beyond the reach of the weaned pigs [**(A)**, left; study 3] and plastic holder of nylon or EVA [**(B)** right; study 4]. The red circle was added to point to the cookie. The plastic holder that contained either nylon or EVA cookie with infused MP is shown on the right photo. Both the cookie and nylon were impregnated with MP prior to placement on the feeders. The configuration of the left photo was also used in Study 4 for the EVA treatment.

Prior to study three, we had not yet performed any sort of dose response study. Therefore, we sought to determine if pigs responded similarly with the concentration used above (the MAME formula) and a formulation that was 5 times higher (0, vs. 1 vs 5 ppm of MP). Study 2 identified that the Myristic Acid formula performed as well as the MAME formula. Rather than the sponge method, study 3 used ethylene vinyl acetate (EVA) plastic beads that were incubated with the pheromone concentrations for 24 h and then melted at 200 C for 11 min. EVA melts at this temperature and the mold we used produced a round cookie-like object (CLO; see Figure 2A). After, the MP-infused CLOs were formed and cooled, they were placed into sealed Whirl-Pak bags until the start of the study. A total of 48 piglets were arranged into 12 pens (4 pens per treatment), randomly distributed to equalize litter, body weight and gender in each treatment. Control pens received a placebo CLO that only held the vehicle (Di isopropyl adipate) with no pheromone. EVA CLOs were mounted to the feed trough, approximately 5 cm above the feed. The CLO's remained above the feed for 7 days before being taken down.

Study 2 found that the MAME results were equivalent to the Myristic Acid formulation. Because of this, study 3 continued research with the Myristic Acid formula. This formula was diluted with di isopropyl adipate instead of 70% isopropyl. The CLOs were made with either a 1 ppm (100 μg) for low dose or 5 ppm for high dose (500 μg). Body weights were collected at time of weaning and 7 days post-weaning. Numbers and percentages of pigs losing weight after weaning were recorded. Again, key measures were ADG at 7 days post-weaning and the percentage of weaned pigs that lost body weight 7 days post-weaning.

#### Study 4—Nylon vs. ethylene vinyl acetate plastic as a delivery device

Products like air fresheners and area insecticides, are sold as an imbedded plastic material that releases the odor or chemical over time. The two most common plastic materials are EVA and Nylon. In this study we sought to determine (using our 7-days bioassay) if one form of plastic or the other would impact pig performance. Study 3 identified the lower concentration (100 μg) to produce similar results as the high dose (500 μg), so Study 4 focused on manufacturing the low dose into strips of Nylon and EVA plastic. The EVA beads (CLO, Figure 2A) were placed above the feed, fastened to the feeder as shown in Figure 2A. The Nylon strips were fabricated and arranged into rectangular plastic holsters and placed on the feeders 5 cm above the feed. Two holder were placed evenly per pen on the feeders (Figure 2B). The holder was opened and the Nylon was inserted.

A total of 60 weaned piglets were arranged by litter and gender into 15 pens (5 pens/treatment). Control pens received nothing. Nylon strips were cut into rectangular pieces (2 cm X 7 cm) and placed into locked plastic holsters with openings on the sides for the release of the pheromone. EVA CLOs, as described above, were suspended above the feed at the same height as the Nylon strips. Nylon strips and EVA cookies remained for 7 days. Weights were recorded at time of weaning and 7 days post-weaning onset. Key measures were ADG the first 7 days post-weaning and the percentage of weaned pigs that lost body weight 7 days post-weaning.

### Meta-analyses of the 7-day bioassay

To run through many iterations of MP delivery requires time, animals, and peoples' efforts. To reduce the time needed to move product development along, a simple, less time-intense bioassay was needed. Individual studies do not have to have significantly different treatments if one is simply interested in which is better among a small number of choices. Here we report one long-term study (study 1) and three bioassay studies (studies 2–4). The methods during the first 7 days of each of the four studies were identical; each study had a control and MP treatment groups. This meta-analysis or experiment-wise examination used the experiment and treatment (control vs. MP regardless of form) as the experimental unit. Each study generates only 1 mean per measure per treatment per study. Therefore, the four studies we summarize here represent an N of 4 experimental units per treatment (the study was the experimental unit). In addition, having the same methodology and the same control treatment over four studies gave us ample sample size to (a) demonstrate overall effects of control vs. MP and (b) estimate *via* power test the sample size needed for a 7-days bioassay to determine that within a single study, treatment effects could be determined. Because in all studies the MP variations (ex., sponge vs. beads) did not differ, here we report the data as control vs. MP (average of 2 treatments that themselves do not differ; in studies 1, 2 and 3 and control vs. Myristic Acid for study 4 because MAME was similar to the control group). Because they did not differ from control pig values, MAME and Nylon treatments were not included in the experiment-wise evaluation of the percentage of pigs losing weight in the first 7 days after weaning.

### Chemical assay for release of MP from EVA or nylon over 48 h

We sought confirmation that the MP was released from the differing plastic forms. Different application methods cause different release rates. Skatole is highly volatile and is used as a marker of MP release. Myristic Acid cannot easily be detected in the head space over the plastics we tested. Solid Phase Microextraction (SPME) was utilized to detect the releasing of the maternal pheromone molecules from the applicators in the study (15). SPME identified the volatile molecules released from the application types through Gas Chromatography Mass Spectrometry (GC-MS). SPME was utilized to mimic the ability of the piglet to sense the pheromone in the air instead of a direct application. The EVA and Nylon plastics were assessed by testing the release of the pheromone from 1 gram of each MP-infused plastic. All samples were incubated with SPME fibers at 90 C for 60 minutes before running through the GC-MS for a retention duration of 60 min. Release levels of the Skatole were measured against a Skatole standard. Both EVA and Nylon samples were tested at 24 h and 48 h post study; the MP-infused plastic was kept in a nursery to simulate on-farm conditions prior to assay.

## Results

### Study 1—Sponge vs. bead MP application

Piglets were placed in one of three treatments: control, sponge-MP, bead-MP. Weights for all treatment groups averaged 5.81 kg at the start of the study with no significant difference between treatment groups and control groups (SE = ± 0.53 kg). Control groups gained 0.04 kg per day while treatment groups gained 0.11 kg per day (*P* < 0.05). For most measures, MP delivered by sponge or bead caused equal behavior and performance. Feeding behavior among pigs with beads-MP was higher (P < 0.05) than sponge-MP during the first 2 h after weaning; but the average feeding behavior over 48 h was not different between sponge-MP and bead-MP. Likewise, for all measures of pig body weight and weight gain, sponge-MP induced similar performance as bead-MP exposed pigs.

Data during the weeks post-weaning are presented in Table 2. Note that ADG during days 0 to 7 after weaning was higher (*P* < 0.05) for sponge-MP and bead-MP compared to the control group. The significant difference of the treatment groups ADG compared to the control suggests that maternal pheromone influences feed intake in newly weaned piglets.

**Table 2:** Least squares means and % difference (Study 1) during the first 7 days after weaning.

	**Body weight, kg**				

**Treatment**	**Weaning**	**7 d**	**ADG, kg/d**	**% Diff**	**% Pigs losing weight**
Control	5.81	6.11	0.04	—	25.0%
Sponge with MP	5.91	6.67	0.11^*^	155%	6.2%^**^
Beads with MP	5.71	6.44	0.10^*^	148%	6.2%^**^
SE	0.53	0.56	0.047	—	—

Body weights until the finishing phase are in Table 3.

MP is Maternal Pheromone. *N* = 4 pens and 16 pigs per mean.g5

* Represents significantly different from control group, P < 0.05.

** While a commercially meaningful difference, the Fisher's exact test P value for MP vs. Control was 0.15 (not statistically significant).

Pig growth was measured into the finishing phase at 16 wk post-weaning. ADG was higher the first week for MP-exposed weaned pigs compared with the control group. Pig body weight at 16 wk post-weaning was higher (*P* = 0.05) among MP-exposed pigs relative to control pigs. The effect is large—MP-exposed pigs were 4 kg heavier than control pigs 16 weeks after weaning. At other weight days (earlier weights- weeks 4 to 12), the means tended to be higher for MP-exposed pigs than control pigs, but none of these other weigh dates reached statistical significance.

Average pig behavior on the first 24 h after weaning is reported in Table 3. Note that lying behavior was very similar for control vs. MP-exposed pigs. However, in mean value, MP tended to cause a reduction in aggression (*P* = 0.06) and an increase in feeding.

**Table 3:** Study 1 pig behavior (over 24 h post-weaning) and performance (weaning until 16 wk later).

	**Treatments**				
**Measure**	**Control**	**MP**	**Difference, %**	**SE**	***P*-Value**
N pigs	16	32	–	–	–
N pens	4	8	–	–	–
***Behavior after weaning, seconds/h/pen over 24 h***
Aggression^*^	84.6	31.8	−62%	17.4	0.06
Feeding^*^	51.7	66.3	+28%	15.4	0.53
Lying	989.7	1035.4	+5%	147.7	0.83
***Performance, kg or kg/d (D 0 is weaning) after weaning***
Weaning	5.81	5.81	0	0.26	0.99
Wk 1	5.94	6.58	+7%	0.24	0.23
Wk 4	15.2	15.79	+4%	0.58	0.50
Wk 8	31.57	33.66	107%	1.24	0.26
Wk 12	49.35	53.25	108%	1.72	0.15
Wk 16	81.96	86.0	105%	1.25	0.05
ADF, 0–16 wk, kg/d	1.69	1.97	117%	0.11	0.11
ADG, d 0–7, kg/d	0.043	0.11	245%	0.022	0.04
ADG, 0–16 wk, kg/d	0.52	0.57	+9%	0.020	0.15
F:G ratio	1.48	1.58	107%	0.057	0.26

* Chi-square of feeding and aggression for Control vs. MP was highly significant (χ^2^ = 20.06, P < 0.01).

**Figure 3:**
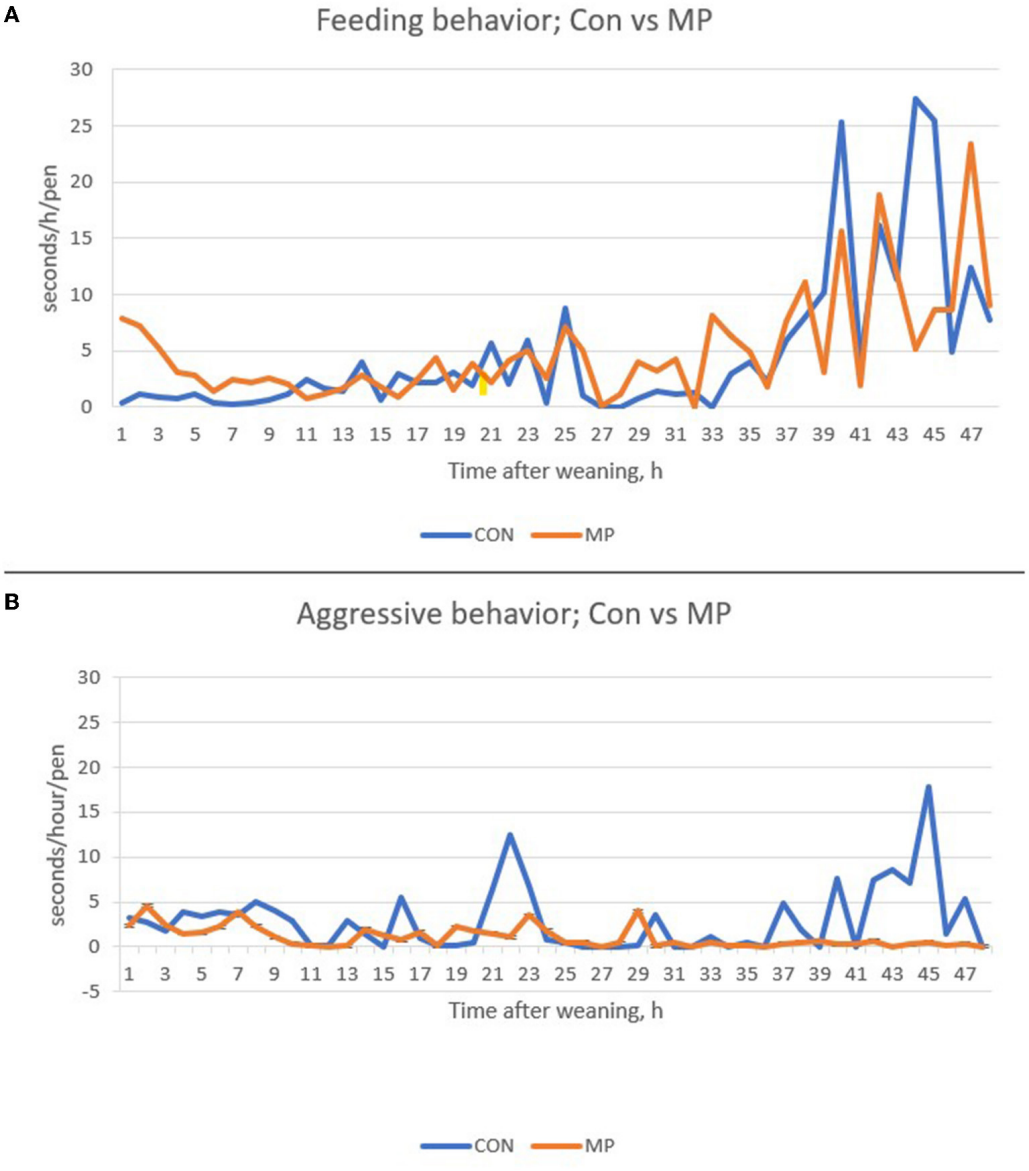
Aggressive behavior **(A)** and feeding behavior **(B)** of pigs in Study 1 that had control (Con) or maternal pheromone (MP) exposure. The MP treatments of Bead and Sponges are combined because they did not differ from each other. Table 2 presents the means for Con and MP treatment groups over the first 24 h after weaning. Note that overall, MP reduced aggression by about 38% and increased feeding behavior about 28% (see Table 3). Lying behavior was not different among treatments. *N* = 4 pens for the control and *N* = 8 pens for the MP treatment. A total of 48 pigs were evaluated. Sep = 1.78 for aggressive behavior and 2.35 for feeding behavior. Within time points, treatments differed for aggressive behavior at hours 23, 40, 42, 43, 45, and 47 h while feeding behavior differed between treatments at time points 1, 2, 40, 44, and 45 h after weaning (*P* < 0.05).

**Figure 4:**
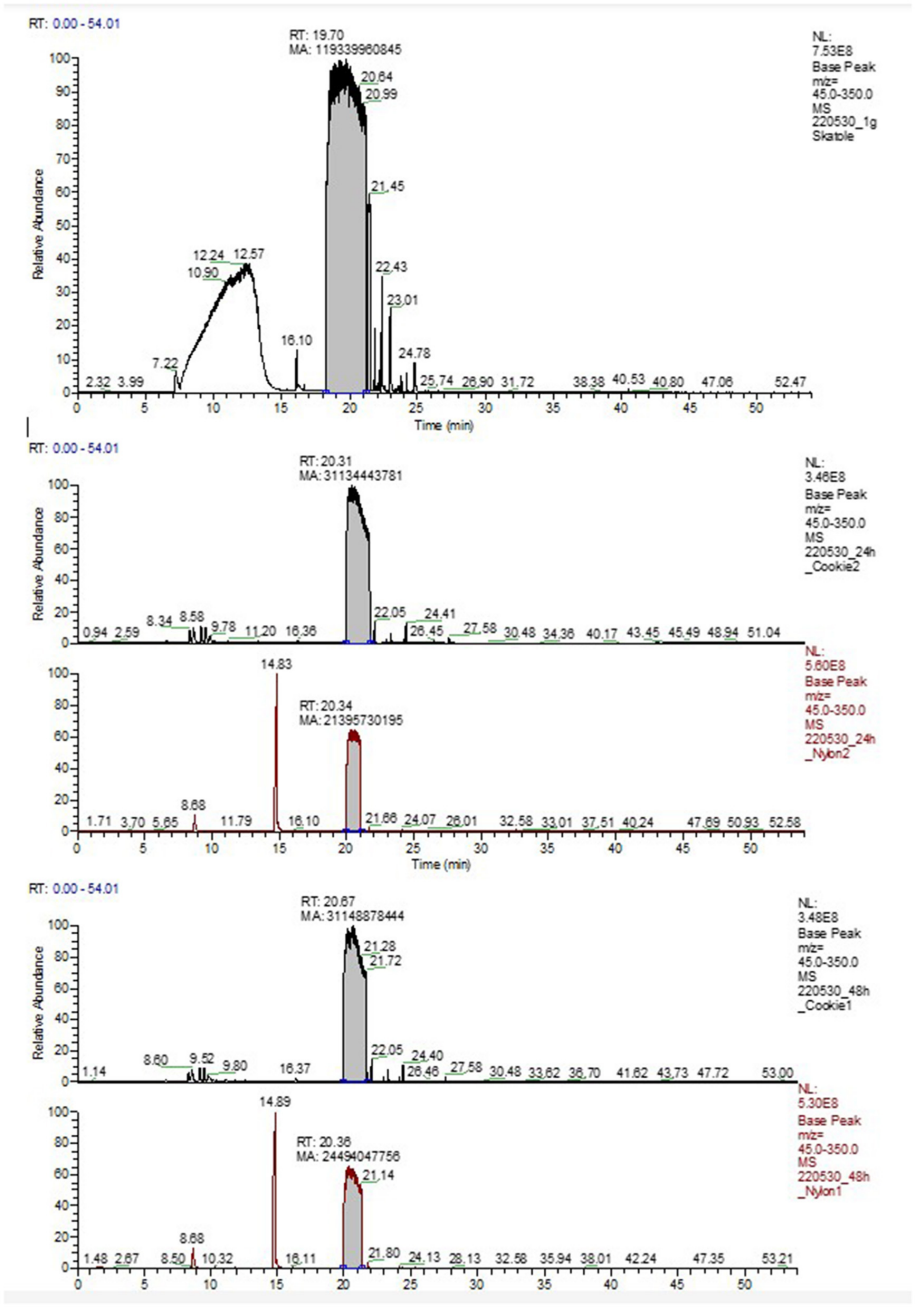
Results of head space analysis over EVA cookies or nylon plastics. Top graph (A) is only skatole (no plastic carrier) which was run as a control sample. Note that Skatole peaks at about 20 min. Other peaks represent unknown molecules that are found in air over the samples. Graphic B represents EVA cookies at 24 h; C represents Nylon skatole release at 24 h; D represents EVA cookie release of skatole at 48 h and E represents nylon skatole release after 48 h. Plastic samples were placed in the university nursery at 32 C as is standard procedure on commercial farms. Graphs are representative samples.

The feeding and aggressive behavior data over the 48 h after weaning are presented in Figures 3A,B. The treatment by time effect was significant, which indicates control and MP-exposed pigs differed in feeding and aggression at some time points (these are given in the figure legend of Figure 4). A 2 X 2 Chi-square calculation of feeding behavior and aggressive behaviors showed that feeding and aggression move in opposite directions (χ*2* = 20.06, *P* < 0.01). That is, when aggression went down, feeding behavior went up.

### Study 2—MAME vs. MA

Study 2 focused on the form of one molecule in the maternal pheromone formula to identify if using MAME might be better than Myristic Acid because MAME is more volatile. Body weights at the beginning of the study were uniform across all pens at an average 6.44 kg. Within this study, the two forms of Myristic Acid did not differ significantly from the control pigs nor from each other (Table 4). However, MAME and Myristic Acid produced nearly identical, small, non-significant increase in body weight and ADG during the first week after weaning. The percentage of pigs that lost weight were 25% for control pigs and 0% for MA-exposed pigs. The MAME treatment group had as many pigs lose weight as the control pigs (25%; Table 4). These results favor use of Myristic Acid over MAME in the formula. Improvement over control group for MA-exposed pigs was not as large in this bioassay as in the other studies.

**Table 4:** Study 2 data showing that CON, MAME, MA did not differ significantly in body weights or ADG in this study.

**Treatment**	**WT d 0, kg**	**WT d 7, kg**	**Avg diff**	**% Diff**	**% pigs losing weight**
Control	6.53	6.96	0.95	–	25%
MA	6.53	7.02	1.09	+15%	0^*^
MAME	6.56	7.06	1.1	+16%	25%

Fewer pigs lost weight with the MA form of the MP.

Means favor the MA and MAME. *N* = 48 pigs; 4 pigs/pen; 3 pens/treatment.

* Pigs exposed to MP in MA form differed from control by Fisher's Exact test, P < 0.05. % Losing body weight was not different comparing control with the MAME form of MP.

### Study 3—Low dose vs. high dose

Low dose and high dose versions were tested in our 7-days bioassay to identify the concentration at which piglets respond to MP exposure. Weights started at an average 6.8 kgs through each treatment group and were compared to weights at 7 days post-weaning. The control group gained 0.02 kg per day with no significant increase in weight due to treatment. Both treatment groups, as seen in study 1 and study 2, demonstrate a significant combined increase in weight of over 50% relative to the control group (Table 5). The low and high dose treatment groups were similar in terms of ADG 7 days post-weaning; however, a large difference was found in the percent of pigs losing weight the first 7 days post-weaning. As in other studies, 25% of control pigs lost body weight the first 7 days post-weaning. Pigs exposed to the low dose of MP had zero pigs losing weight the first week post-weaning while the higher dose of MP had 25% of pigs losing weight the first week post-weaning. While largely not different, the results favor the lower dose to capture some increase in body weight and fewer pigs losing weight after weaning.

**Table 5:** Dose response for MP in EVA cookies (Study 3).

	**Body wt, kg**				
**Treatments μg MP/batch**	**Weaning**	**D 7**	**ADG**	**% Diff**	**% Losing body weight**
0	6.86	7.04	0.026	–	25%
100	6.90	7.21	0.043	+65%	0%^**^
500	6.73	7.10	0.053^*^	+104%	25%
SE	0.24	0.25	0.016	–	–

*N* = 12 pens and 3 pens per treatment.

* Trend for different from Control, P = 0.10.

** Pigs exposed to 100 μg dose differed from control by Fisher's Exact test, P < 0.05. % Losing body weight was not different comparing control with the 500 μg dose of MP.

### Study 4—EVA vs. nylon plastic

This study evaluated two forms of plastic that are known to be used to release volatile molecules (such as air fresheners, insecticides, and pheromones). Pigs in treatments exposed to either the EVA or Nylon had an increase in ADG the first 7 days post-weaning, but the effects were not statistically significant (Table 6). Once again, the 25% of control pigs lost weight after weaning. Pigs exposed to EVA-MP had zero pigs losing weight the first week after weaning (P < 0.01 relative the control). Pigs exposed to Nylon-MP had 25% of pigs losing body weight the first week after weaning (not different from control, but different from the EVA-MP treatment group). While both forms of plastic more than doubled ADG the first 7 days post-weaning, the fact that EVA-MP exposed piglets had no pigs that lost body weight favors use of EVA over Nylon as a carrier of this pheromone.

**Table 6:** Pig performance for MP in EVA cookies vs. Nylon (Study 4).

	**Body wt, kg**				
**Treatments μg MP/batch**	**Weaning**	**D 7**	**ADG**	**% Diff**	**% Losing body weight**
Control	6.9	7.0	0.026	–	25%
EVA	6.8	7.2	0.058	+123%	0%^*^
Nylon	6.7	7.1	0.063	+142%	25%
SE	0.52	0.55	0.040	–	–

N = 12 pens and 3 pens per treatment.

* Pigs exposed to EVA-MP differed from control by Fisher's Exact test, P < 0.05. % Losing body weight was not different comparing control with the Nylon-MP. Other measures (body weights and ADG) did not differ significantly among treatments.

### Chemistry

**Figure 5:**
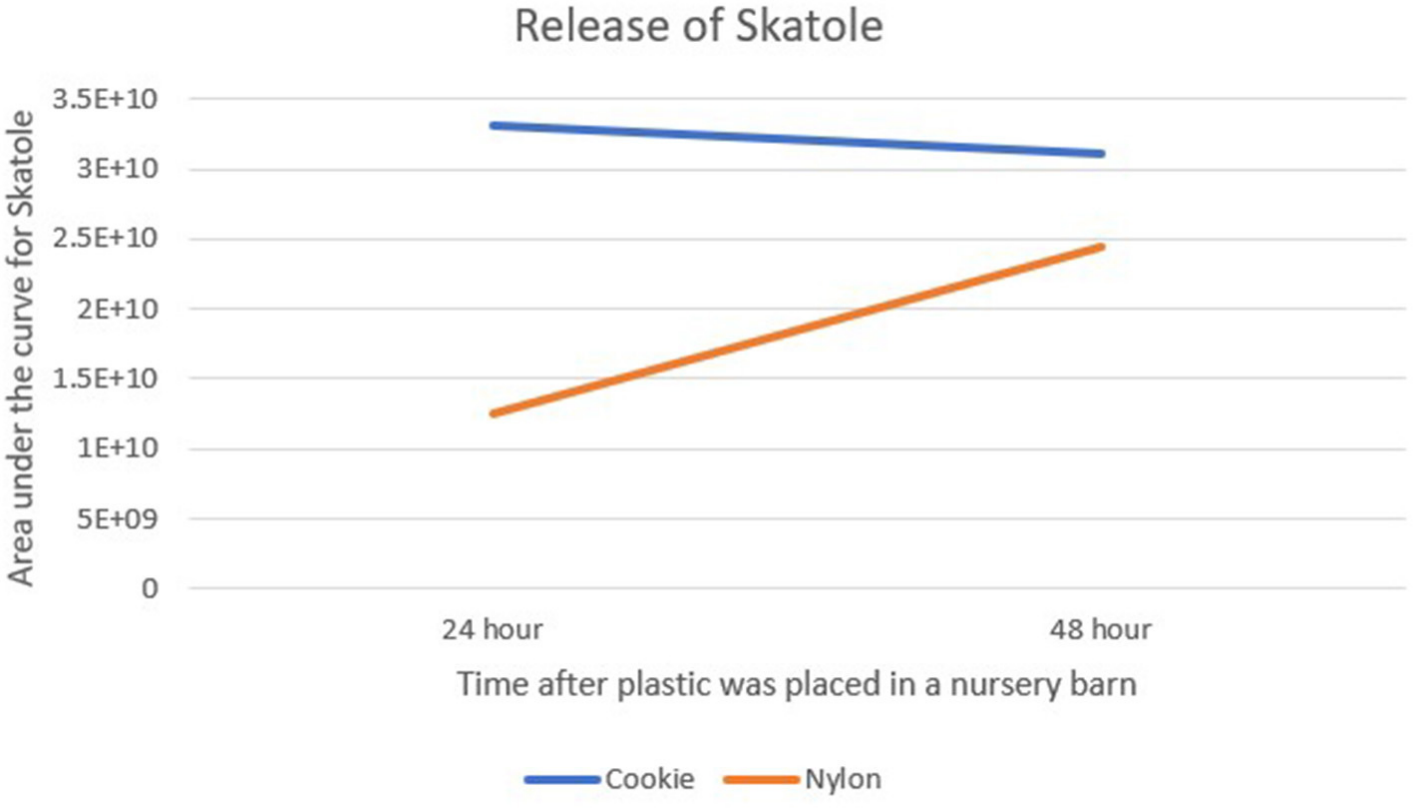
Area under the curve for Skatole measured in the head space over EVA cookies or Nylon plastic then subjected to GC-MS analyses. Note that EVA cookies were higher than skatole release from nylon. Also, while EVA cookies were fairly stable over time, the nylon released more skatole over time (from 24 to 48 h). Pig performance data support EVA cookies as more effective than skatole released from nylon. These are a representative single sample of each time point and plastic type.

Comparing the GC-MS of each group, EVA released more MP than the Nylon (Figures 4, 5). Comparing release levels over 24 and 48 h post weaning time, EVA-MP plastic had an increased area under the curve of pheromone compared to the Nylon-MP. Interestingly, the EVA plastic was fairly stable over the 48 h, but still consistently higher than Nylon, and skatole in the Nylon increased release over the 48-h period. This may explain the results identified in Study 4 and why the EVA plastic material provided high performance averages (fewer pigs losing weight) from piglets in each study. The Nylon plastic increased its release rate over time, so the piglet was not exposed to the pheromone as intensely as the EVA at time of weaning. The immediate impact of the pheromone at time of weaning is what seems to allow the piglet to have a greater success of survival and increased performance.

## Discussion

Study 1 and the meta-analyses demonstrate that the MP was efficacious at improving pig post-weaning weight gain and the MP prevented many pigs from losing weight after weaning in general. The maternal pheromone induced this performance stimulation by increasing feeding behavior and reducing aggressive behaviors in the post-weaning environment. The responses we obtained were larger than our previous study (5). Previously, we used a ratio of Skatole to Myristic Acid of 2:1; however, we now see that the ratio in biological samples is closer to 1:1. Our studies here used the new biologically correct ratio (the one closer to what sows emit). In study 1, feeding behavior was measured, a much greater response with the 1:1 ration was observed compared with our earlier response of about 30% increase in feeding behavior. In general, this current work replicates and extends the earlier studies (5).

We observed zero mortality in these studies. This would not be the case on most commercial farms. This work needs to be replicated and perhaps refined for an actual commercial environment which represents normal pig production.

Feed consumption is low in the recently weaned pig and is a current challenge (2). The data presented in Figure 3A shows that control pigs did not eat well for 12 to15 h post- weaning. Pigs exposed to the MP ate in the first hour—this is generally not observed on commercial farms. Finding the feeder and feed and eating in the first few hours after weaning has a positive effect on pig health and performance that lasts months. This is exemplified in our first study, where pigs exposed to MP for just the first 2 days were over 4 kg heavier than control pigs 16 weeks later. This effect is larger than many pharmaceutical products. Pheromones are not drugs according to the United States FDA and can be marketed as clean, green ethical technologies that improve pig performance and welfare.

The 7-days bioassay accelerated our refinement of the best delivery options to get the MP near the pigs (Table 7). The model assesses two key measures. Pig ADG after weaning is often very low and quite variable the first week. Often, weaned pigs are about the same weight 7 days post-weaning as they were at weaning (2). This is because some weaned pigs lose weight, and some pigs gain a small amount of weight that first week. The percentage of pigs that lost weight over 7 days post-weaning is a less variable and highly sensitive measure of pig performance and welfare. We produced data from 4 studies that show this 7-day bioassay can provide meaningful information on dose and form of delivery. In three of the four bioassays, at least one form of MP caused zero pigs to lose weight post-weaning. This, of course, increased the average weight and weight gain of the group. In study 1, pigs losing weight went from 25 to 6% with the MP and while this is in the same direction as other studies, it did not reach statistical significance (Table 2).

**Table 7:** Results of ADG and % of pigs losing weight from weaning until 7 days later over 4 studies.

					**% pigs losing weight** ^***^	
**Study**	**Item**	**CON**	MP^*^	**% Difference**	**CON**	**MP**
	N Pigs	48	40			
1	Sponge & Beads vs. CON	0.093	0.234	+152%	25.0%	6%
2	MAME & MA vs. CON	0.135	0.157	+16%	25.0%	0%
3	100 μg dose vs. CON	0.052	0.121	+133%	25.0%	0%
4	EVA vs. CON	0.026	0.058	+123%	25.0%	0%
	Average	0.118	0.184	+56%	25.0%	1.5%
	SE	0.018			0	1.3
	*P*-value for effect	0.08			0.001	
Number of replicates needed for a 200% change (doubling)^**^		23				

Each study's means are shown here and, using the experiment as the experimental unit, an overall effect showed a trend (*P* = 0.08) for the MP to stimulate post-weaning ADG. Given the large variation in post-weaning performance, a power test revealed that 23 replicate pens are needed per treatment to detect a large (doubling) effect on ADG. MP-exposed pigs had fewer pigs losing weight the first week than control pigs.

* MP treatments were combined or one treatment (if not changed) was deleted in this analysis because the different methods of delivery did not cause a meaningful change in post-weaning ADG, except for (Studies 2 and 4). MP infused into Nylon and MAME were not different from CON values.

** A power test indicated that, if the 0 to7 day post-weaning ADG more than doubled (as in Studies 1 and 3), that an N of 23 pens would be needed to detect a 150% increase in ADG with P < 0.05 and alpha = 0.80.

*** Percentage of pigs losing weight was higher (P < 0.01) overall among control pigs than MP-treated pigs. Less effective MP treatments were not included in this summary (Nylon and 500 μg dose of MP).

Clearly, weaned pigs that lose weight have poor welfare. Preventing that loss in body weight will improve pig welfare, as pigs that lose weight are believed to be more susceptible to disease and starvation. The MP may give the weaned pig an olfactory clue as to where the feed is and encourage them to eat. When feeding behavior goes up, aggression goes down (they cannot express both behaviors at the same time). Reduction in aggression at this age does not have a large economic impact, but it is positive for pig welfare. Moreover, increasing feeding behavior has pig performance and health benefits.

We can now make some general conclusions about use of the MP to stimulate post-weaning performance and behavior. First, the effective dose is fairly wide. Although 1 ppm and 5 ppm gave similar results, we also recognize that the sponge, alginate beads, and EVA each release MP at different rates, yet they were all largely effective.

One could ask why we do not put the MP in the feed. First, that would require regulatory approval which takes time, money, and may not be necessary if non-feed applications are effective. Second, we are trying to get the weaned pig to understand that this dry grain-based substance is its food rather than milk from its mother. Having the maternal odor near, but not in the food, gives the piglet the idea that food is in proximity of the MP. We would not recommend spraying the MP anywhere other than near the feed in the feeder. If one sprayed it all over the post-weaning pen, it would confuse the weaned pigs much like spraying pheromones in a forest disrupts mating behavior by confusing the insects and preventing insect mating (16). The immediate postweaning influence will dictate the improvement or decline of the piglet. By means of the EVA plastic, the MP was released at high, consistent levels instantly and uniformly over 48 h. This allows the piglet to have greater chance of success, because of the instant positive influence from the pheromone during the time of weaning.

Another indication that the effect is robust is that the MP has positive effects when delivered in many forms. Besides the many forms tested here, Iowa State Scientists had positive results diluting the MP in sunflower oil (11)and our original work was in mineral oil (5). Then, we used isopropyl alcohol and di isopropyl adipate as the vehicles—all with positive results. Therefore, the effective dose and form of delivery are robust in their variations that can reliably produce results.

One can see about a 4 kg body weight advantage when the MP was used for 2 days after weaning. Few animal health products deliver this level of response. By considering that the post-weaning environment is deficient in the MP, adding this pheromone back into the weaning space restores greater (or more normal) feed intake, produces less fighting, and causes greater weight gain. Producing both better performance and welfare.

The MP has many applications beyond what is reported here. It is useful to attract pigs of any age to a device or food, and it may reduce stress when the pigs experience stressors like weaning or transport. Future studies will examine how to correct the olfactory environment present in production settings, known to be deficient in biologically relevant semiochemicals, as a means to stimulate health, productivity, and welfare.

## Data availability statement

The original contributions presented in the study are included in the article/Supplementary material, further inquiries can be directed to the corresponding author.

## Ethics statement

The animal study was reviewed and approved by Texas Tech University Animal Care and Use Committee (# 19104-12).

## Author contributions

JM conceived the project and supervised the studies. CA conducted most of the work. AG provided the technology for the alginate beads. MH assisted in the writing of the manuscript and its preparation for publication. All authors contributed to the article and approved the submitted version.

## Funding

This work was supported by Animal Biotech, LLC which provided funds to Texas Tech University for the conduct of this work.

## Conflict of interest

JM declares a potential conflict of interest in that he is the inventor and patent-holder of the MP technology reported here. The intellectual property is owned by Texas Tech University who licensed this technology to Animal Biotech, LLC.

The remaining authors declare that the research was conducted in the absence of any commercial or financial relationships that could be construed as a potential conflict of interest.

## Publisher's note

All claims expressed in this article are solely those of the authors and do not necessarily represent those of their affiliated organizations, or those of the publisher, the editors and the reviewers. Any product that may be evaluated in this article, or claim that may be made by its manufacturer, is not guaranteed or endorsed by the publisher.

## Abbreviations

CLO: Cookie like Object
EVA: Ethylene Vinyl Acetate
GC-MS: Gas Chromatography Mass Spectrometry
MA: Myristic Acid
MAME: Myristic Acid Methyl Ester
MP: Maternal Pheromone
SPME: Solid Phase Microextraction.
